# Determinants for Achieving the LDL-C Target of Lipid Control for Secondary Prevention of Cardiovascular Events in Taiwan

**DOI:** 10.1371/journal.pone.0116513

**Published:** 2015-03-10

**Authors:** Li-Ting Ho, Wei-Hsian Yin, Shao-Yuan Chuang, Wei-Kung Tseng, Yen-Wen Wu, I-Chang Hsieh, Tsung-Hsien Lin, Yi-Heng Li, Lien-Chi Huang, Kuo-Yang Wang, Kwo-Chang Ueng, Ching-Chang Fang, Wen-Harn Pan, Hung-I Yeh, Chau-Chung Wu, Jaw-Wen Chen, Taiwanese Secondary Prevention for patients with AtheRosCLErotic disease (T-SPARCLE) Registry Investigators

**Affiliations:** 1 Division of Cardiology, Department of Internal Medicine, National Taiwan University College of Medicine and Hospital, Taipei, Taiwan; 2 Division of Cardiology, Heart Center, Cheng-Hsin General Hospital, and School of Medicine, National Yang-Ming University, Taipei, Taiwan; 3 Institute of Population Health Sciences, National Health Research Institutes, Miaoli, Taiwan; 4 Department of Medical Imaging and Radiological Sciences, I-Shou University and Division of Cardiology, Department of Internal Medicine, E-Da Hospital, Kaohsiung, Taiwan; 5 Cardiology Division of Cardiovascular Medical Center and Department of Nuclear Medicine, Far Eastern Memorial Hospital, New Taipei City, Taiwan; 6 Second Department of Cardiology, Chang-Gung Memorial Hospital, New Taipei City, Taiwan; 7 Division of Cardiology, Department of Internal Medicine, Kaohsiung Medical University Hospital and Kaohsiung Medical University, Kaohsiung, Taiwan; 8 Department of Internal Medicine, National Cheng Kung University Hospital and College of Medicine, Tainan, Taiwan; 9 Department of Cardiology, Taipei Union Hospital, Taipei, Taiwan; 10 Cardiovascular Center, Taichung Veterans General Hospital and Department of Medicine, Chung-Shan Medical University, Taichung, Taiwan; 11 School of Medicine, Chung Shan Medical University and Department of Internal Medicine, Chung Shan Medical University Hospital, Taichung, Taiwan; 12 Division of Cardiology, Department of Internal Medicine, Tainan Municipal Hospital, Tainan, Taiwan; 13 Mackay Memorial Hospital, Mackay Medical College, New Taipei City, Taiwan; 14 Department of Primary Care Medicine, College of Medicine, National Taiwan University, Taipei, Taiwan; 15 Department of Medical Research and Education, Taipei Veterans General Hospital and Institute of Pharmacology, National Yang-Ming University, Taipei, Taiwan; Osaka University Graduate School of Medicine, JAPAN

## Abstract

**Background:**

Epidemiological and clinical studies have clearly established the link between low-density lipoprotein cholesterol (LDL-C) and atherosclerosis-related cardiovascular consequences. Although it has been a common practice for physicians to prescribe lipid-lowering therapy for patients with dyslipidemia, the achievement rate is still not satisfied in Taiwan. Therefore, the determinants for achieving the LDL-C target needed to be clarified for better healthcare of the patients with dyslipidemia.

**Method:**

This registry-type prospective observational study enrolled the patients with cardiovascular diseases (coronary artery disease (CAD) and cerebrovascular disease (CVD)) from 18 medical centers across Taiwan, and clinically followed them for five years. At every clinical visit, vital signs, clinical endpoints, adverse events, concurrent medications and laboratory specimens were obtained as thoroughly as possible. The lipid profile (total cholesterol, high-density lipoprotein cholesterol, LDL-C, triglyceride), liver enzymes, and creatinine phosphokinase were evaluated at baseline, and every year thereafter. The cross sectional observational data was analyzed for this report.

**Result:**

Among the 3,486 registered patients, 54% had their LDL-C < 100 mg/dL. By univariate analysis, the patients achieving the LDL-C target were associated with older age, more male sex, taller height, lower blood pressure, more under lipid-lowering therapy, more smoking cessation, more history of CAD, DM, physical activity, but less history of CVD. The multivariate analysis showed statin therapy was the most significant independent determinant for achieving the treatment target, followed by age, history of CAD, diabetes, blood pressure, and sex. However, most patients were on regimens of very-low to low equipotent doses of statins.

**Conclusion:**

Although the lipid treatment guideline adherence is improving in recent years, only 54% of the patients with cardiovascular diseases have achieved their LDL-C target in Taiwan, and the most significant determinant for this was statin therapy.

## Introduction

Cardiovascular disease, including coronary artery disease (CAD) and cerebrovascular disease (CVD), is common in the general population, especially in adults past the age of 60 years. In 2012, cardiovascular disease was estimated to result in 17.3 million deaths worldwide on an annual basis [1]. Atherosclerosis is responsible for almost all cases of cardiovascular diseases, especially CAD. A variety of factors are associated with an increased risk for atherosclerosis, including age, family history, current cigarette smoking, hypertension, diabetes and dyslipidemia.

Twenty-five year follow-up data from the Seven Countries study show that serum total cholesterol (TC) levels are linearly related to CAD mortality across cultures [2]. The link between high cholesterol levels and increased incidence of cardiovascular disease has also been shown in the prospective part of the Multiple Risk Intervention study [3]. In epidemiological studies, measurements of serum cholesterol have been routinely used. Besides, high LDL-cholesterol (LDL-C) level is a particularly important risk factor for atherosclerosis [4,5], and has been associated with an increased incidence of CAD in a large number of studies [6]. Therefore, LDL-C has long been identified by NCEP as the primary target of cholesterol-lowering therapy. In 2004 updated NCEP ATP III and 2006 updated ACC/AHA guidelines, LDL-C should be <100 mg/dL for all patients with CAD or CAD risk equivalents, but in addition, it is reasonable to lower LDL-C to <70 mg/dL in such patients with very high risk [7,8].

Although it has been a common practice for physicians to prescribe lipid-lowering therapy for patients with dyslipidemia, the achievement rate is still not satisfied in the real world [9,10]. In the REALITY-Asia study, only 38% of high risk patients attained ATP III targets for LDL-C (<100mg/dL) in Asians [11]. Although there is a well-established national medical insurance system in Taiwan, the LDL-C goal attainment percentage is still low in those high-risk patients. Therefore, the determinants for achieving the LDL-C target needed to be clarified for better healthcare of the CVD patients.

## Method

### 2.1 Study population

This study was conducted from a multi-center observational registry, the Taiwanese Secondary Prevention for patients with AtheRosCLErotic disease (T-SPARCLE) Registry, from 14 teaching hospitals in Taiwan [12,13]. This registry attempts to recruit and follow-up a large population of patients with cardiovascular diseases who have been receiving secondary prevention therapies so as to define the current status of these therapies and their effects on morbidity and mortality in Taiwan.

Adult patients (>18 year-old) who had stable cardiovascular diseases, including CAD and CVD, were recruited. Patients with CAD was defined as those who had significant coronary artery stenosis (>50%), or had a history of myocardial infarction, or who had angina showing ischemic electrocardiographic changes or positive response to stress tests. Patents with CVD were defined as those with cerebral infarction, intra-cerebral hemorrhage, transient ischemic attack attributed to cervical or intracranial large artery stenosis (>50%). Patients with neurocognitive or psychiatric condition, life expectancy of less than 6 months, and hemodynamically significant valvular or congenital heart disease are excluded.

### 2.2. Ethic statement

The study was approved by the Joint Institutional Review Board, Taiwan, R.O.C. for each participating hospital. The JIRB number was 09-S-015. Written informed consents were obtained from all patients.

### 2.3 Targets measurement

Eligible patients who fulfilled the enrolment criteria would be followed up every year for a total of 5 years. At every clinic visit, vital signs, clinical endpoints, adverse events, concurrent medications and laboratory specimens were obtained as thoroughly as possible. The lipid profiles (TC, HDL-C, LDL-C, and TG), liver enzymes, and creatinine phosphokinase were evaluated at baseline, and every year thereafter. The concurrent medications and their dosage were recorded in detail, especially the lipid-lowering drugs (e.g., statin, fibrate, ezetimibe, bile acid sequestrants, nicotinic acid).

The Taiwanese guideline recommended lipid target was applied for evaluation of the achievement. The optimal LDL-C level was <100mg/dL.

### 2.4 Statistical analysis

Categorical variables are presented as percentage and continuous or discrete variables as mean ± standard deviation. The χ2 test was used to compare proportions; student’s t test or analysis of variance was applied to compare difference in continuous variables between groups. A logistic regression analysis was adapted to evaluate the odds ratio and 95% confidence intervals (CI) of the recommended lipid target. Statistical analyses were performed using the SPSS software package version 17.0 (SPSS Inc., Chicago, IL).

## Result

From January, 2010 to February, 2011, 4561 patients were enrolled and 3486 patients (men, 68.4%; female, 31.6%; mean age, 65.8 ± 12 years) included in this analysis. Of these, 2163 (62.1%) had CAD; 921 (26.4%) had family history of premature CAD; 604 (17.3%) had previous stroke or TIA history. The demographics and clinical characteristics of the patients are shown in Table 1. Only 54% of the patients achieved the optimal LDL-C level (<100mg/dL); 69.1% achieved the HDL-C goal (>40mg/dL); 31.1% achieved optimal TG level (<150mg/dL).

**Table 1:** Patients’ demographics and clinical characteristic.

Variable	N	Mean	STD
Age, yrs	3486	65.79	11.96
Male, %	2386	68.45%	
Waist, cm	3110	93.53	11.08
Hip, cm	3034	100.60	8.74
W/H ratio	3038	0.93	0.11
Height, cm	3329	162.69	8.31
Weight, kg	3357	69.73	12.51
BMI, kg/m^2^	3303	26.25	4.08
Hypertension	2611	74.9	
SBP, mmHg	3264	132.60	17.36
DBP, mmHg	3240	76.14	11.10
Pulse rate, beats/min	2607	74.80	12.80
Current Smoker	487	14	
Physical Activity <3times/w, %	1905	54.6%	
TC, mg/dL	3486	174.79	40.88
HDL-C, mg/dL	3486	45.69	14.11
Low HDL <40mg/dL, %	1075	30.8%	
LDL-C, mg/dL	3486	101.47	34.48
High LDL-C >100mg/dL, %	1604	46%	
TG, mg/dL	3486	139.95	90.04
High TG >200mg/dL, %	2401	68.9%	
Creatinine, mg/dL	3057	1.13	0.74
AC Sugar, mg/dL	3080	118.36	40.98
HBA1C, %	1750	7.29%	4.68
AST, mg/dL	1917	28.99	17.94
ALT, mg/dL	2695	29.00	20.27
CK, mg/dL	1451	132.14	303.98

STD, standard deviation; W/H ratio, weight and height ratio; SBP, systolic blood pressure; DBP, diastolic blood pressure; TC, total cholesterol; HDL-C, high density lipoprotein cholesterol; LDL-C, low density lipoprotein cholesterol; TG, triglyceride; AST, aspartate aminotransferase; ALT, alanine aminotransferase; CK, creatinine kinase.

Among these patients, 2434 (69.8%) had medical treatment for dyslipidemia. About 89.8% of the treated patients were on monotherapy with statin or other lipid-lowering medication. The details of lipid-lowering treatment are shown in Table 2. Most patients were on regimens of very low (<1 dose/day, 23%) to low (1–1.9 dose/day, 38%) equipotency doses of statins [14]. The statin potency comparison is listed in Table 3.

**Table 2:** Treatment to the target by drugs.

Achieved lipid level (mg/dL)	N, %	LDL-C<100	TC<160	TG<150	HDL-C≥40/50
No lipid lowering drugs	1052, 30.2%	44.96%	31.75%	71.01%	51.43%
With lipid lowering drugs	2434, 69.8%				
Monotherapy	2185, 89.8%				
Statin	2040	58.82%	44.31%	69.71%	52.55%
Fibrate	131	50.38%	25.95%	25.95%	25.19%
Ezetimibe or Cholestyramine (E or C)	13	23.08%	15.38%	61.54%	53.85%
Nicotinic acid (N)	1	0%	0%	0%	0%
Two-combined therapy	241, 9.9%				
Statin + Fibrate	63	65.08%	36.51%	41.27%	31.75%
Statin + E or C	168	51.79%	39.88%	63.10%	58.93%
Statin + N	6	83.33%	16.67%	16.67%	0%
Fibrate + E or C	3	66.67%	33.33%	33.33%	66.67%
Fibrate + N	1	100%	0%	0%	0%
Three-combined therapy	8, 0.3%				
Statin + Fibrate + E or C	8	50%	0%	12.50%	25.00%

TC, total cholesterol; HDL-C, high density lipoprotein cholesterol; LDL-C, low density lipoprotein cholesterol; TG, triglyceride.

**Table 3:** Statin equipotency.

Equipotent doses	Lovastatin	Pravastatin	Simvastatin	Fluvastatin	Atorvastatin	Rosuvastatin
1 Dose	20 mg	20 mg	10 mg	40 mg	5 mg	2.5 mg
2 Doses	40 mg	40 mg	20 mg	80 mg	10 mg	5 mg
4 Doses	80 mg	80 mg	40 mg		20 mg	10 mg
8 Doses			80 mg		40 mg	20 mg

By univariate analysis, the patients achieving the LDL-C target were associated with older age, more male sex, taller height, lower blood pressure, lower baseline cholesterol levels, more smoking cessation, more history of CAD, DM, physical activity, but less history of stroke or TIA (Table 4).

**Table 4:** Difference of clinical characteristics between the patients achieving and not achieving LDL-C target.

	LDL-C < 100 mg/dL			LDL-C ≥ 100 mg/dL			
Variable	N	Mean	Std	N	Mean	Std	p-value
Age, yrs	1882	65.93	11.65	1604	65.63	12.31	0.4728
Men,%	1330	70.67%		1056	65.84%		0.0022
Waist, cm	1676	93.6	11.12	1434	93.44	11.03	0.6885
Hip, cm	1644	100.62	8.36	1390	100.57	9.16	0.8793
W/H ratio	1637	0.93	0.11	1401	0.93	0.12	0.7862
Height, cm	1784	163.03	8.24	1545	162.29	8.37	0.0106
Weight, kg	1801	69.61	12.3	1556	69.86	12.75	0.571
BMI, kg/m^2^	1784	26.35	4.27	1519	26.14	3.85	0.1272
SBP, mmHg	1752	131.68	17.43	1512	133.66	17.23	0.0012
DBP, mmHg	1740	75.12	11.03	1500	77.33	11.06	<0.0001
Pulse rate, beats/min	1338	74.5	12.76	1269	75.13	12.85	0.2116
TC, mg/dL	1882	149.85	25.62	1604	204.05	35.7	<0.0001
HDL-C, mg/dL	1882	45.07	14.67	1604	46.43	13.38	0.0042
LDL-C, mg/dL	1882	77.04	15.45	1604	130.13	27.99	<0.0001
TG, mg/dL	1882	133.89	99.51	1604	147.05	77.44	<0.0001
Creatinine, mg/dL	1681	1.15	0.8	1376	1.11	0.66	0.1252
AC sugar, mg/dL	1708	118.24	41.27	1372	118.51	40.63	0.8576
HBA1C, %	1014	7.43	5.95	736	7.11	1.82	0.1012
AST, mg/dL	1096	25.85	16.91	821	29.17	19.24	0.7057
ALT, mg/dL	1514	28.51	19.82	1181	29.69	20.82	0.1513
CK, mg/dL	883	126.79	252.66	568	140.47	370	0.4397

Questionnaire	N	%	N	%	p-value
Smoking-Never	1216	64.61	1045	65.15	0.1375
-Current	248	37.24	239	42.75	—
-Cessation	418	62.76	320	57.25	—
Family history of MI/Sudden Death/CVD	510	32.44	411	31.14	0.4526
Family history of diabetes	410	26.45	354	26.84	0.8153
History of hypertension	1399	76.74	1212	78.19	0.3149
History of Heart failure	168	9.8	124	8.81	0.3454
History of MI or CAD	1274	74.27	887	63.18	<0.0001
History of DM, IFG, or IGT	796	45.12	595	40.73	0.012
History of ischemic stroke	197	11.43	214	15.19	0.002
History of non-ischemic stroke	40	2.36	40	2.88	0.3711
History of TIA	46	2.72	67	4.82	0.002
Alcohol consumption	243	12.96	211	13.17	0.8539
Physical activity habits	890	47.29	691	43.11	0.0134

W/H ratio, weight and height ratio; SBP, systolic blood pressure; DBP, diastolic blood pressure; TC, total cholesterol; HDL-C, high density lipoprotein cholesterol; LDL-C, low density lipoprotein cholesterol; TG, triglyceride; AST, aspartate aminotransferase; ALT, alanine aminotransferase; CK, creatinine kinase; MI, myocardial infarction; CVD, cerebrovascular disease; CAD, coronary artery disease; DM, diabetes mellitus; IFG, impaired fasting glucose; IGT, impaired glucose tolerance; TIA, transient ischemic attack.

The multivariate analysis showed statin therapy was the most significant independent determinant for achieving the treatment target (odd ratio 1.53, p-value <0.0001), followed by age, history of CAD, DM, controlled blood pressure, and sex. (Table 5)

**Table 5:** Determinants for achieving LDL-C target by multivariate analysis.

	β	Odds ratio	95% confidence interval	p-value
Age				
<65 years		1.00	—	—
65–74 years	0.278	1.321	1.10–1.59	0.0033
>75 years	0.399	1.49	1.22–1.83	0.0001
Sex: Male vs Female	0.246	1.279	1.06–1.55	0.0111
BMI				
<24		1.00	—	—
24–26.9	-0.118	0.889	0.73–1.08	0.2428
> = 27	-0.160	0.852	0.70–1.04	0.1085
Hypertension				
SBP> = 140 or DBP> = 90	-0.221	0.802	0.68–0.95	0.0086
SBP<140 and DBP<90		1.00	—	—
Smoking: Cessation vs Current	-0.025	0.975	0.81–1.17	0.7846
History of MI or CAD: Yes vs No	0.309	1.362	1.13–1.64	0.0011
History of DM, IFG, or IGT: Yes vs No	0.247	1.279	1.09–1.50	0.0023
History of CVD: Yes vs No	-0.213	0.808	0.65–1.01	0.0581
Physical activity: Yes vs No	0.070	1.073	0.92–1.26	0.3854
Statin: Yes vs No	0.425	1.53	1.29–1.81	<0.0001
Fibrate: Yes vs No	0.173	1.188	0.85–1.66	0.3149

BMI, body mass index; SBP, systolic blood pressure; DBP, diastolic blood pressure; MI, myocardial infarction; CAD, coronary artery disease; DM, diabetes mellitus; IFG, impaired fasting glucose; IGT, impaired glucose tolerance; CVD, cerebrovascular disease.

## Discussion

It is well known that adequate control of dyslipidemia is important in both primary and secondary prevention of cardiovascular diseases. It has been a common practice for physicians to prescribe lipid-lowering therapy for patients with dyslipidemia and the use of lipid-lowering agents has increased in the recent years. However, there is still a large treatment gap between guideline recommendation and the real-world lipid target achievement although there has been a well-established national medical insurance system in Taiwan.

In 2009, Kornelia, K., et al. [15] compared the results of EUROASPIRE I, II and III surveys [16–18], which conducted in European countries and enrolled patients who had CAD and underwent coronary artery bypass graft surgery or percutaneous transluminal coronary angioplasty, or had acute myocardial infarction history. The proportion of patients with raised blood cholesterol concentration (>4.5mmol/L, 174mg/dL, set by the 2003 Joint European Societies’ guidelines) was lowest in the EUROASPIRE III, and highest in the EUROASPIRE I. That means the lipid target achievement rate has improved over time. Therapeutic control in those taking lipid-lowering drugs has also improved. However, the lipid target achievement rate in EUROASPIRE III was only 53.8%, which is still not satisfied.

In the United States, Jones, P.H., R. Nair, and K.M. Thakker [19] reported a cross-sectional, retrospective study of 3 data sources among 2003–2010, which showed, 67% to 77% achieved LDL-C <100 mg/dL in high-risk patients treated with statin monotherapy for >90 days. In contrast in Asia, the REALITY-Asia study published in 2008 [11], which enrolled 2622 patients from 6 Asian countries, showed only 38% of those with CAD/diabetes attained the ATP III targets for LDL-C (<100 mg/dL). The target achievement rate was lower than those of the Western countries. Among these Asian countries, goal achievement rate in Taiwan was the lowest (16%) in the CAD/diabetes group. In the present study, the target achievement rate was 54%, which is much higher than the result of REALITY-Asia, and is similar to the result of EUROASPIRE III. That means the physicians in Taiwan have paid more attention on the lipid control for the cardiovascular diseases secondary prevention in recent years.

The multivariate analysis in this study showed statin therapy was the most significant independent determinant for achieving the treatment target. However, most of the patients had low and very low equipotent doses of statins. From 2003to 2005, we conducted an unpublished retrospective study to survey the physicians’ behaviour on statin usage. About 66% physicians would not modify their prescription even when the treatment target was not achieved at the starting dose of the drugs, especially among the physicians working in the local area hospitals. The most common (32%) rationale for this decision was they thought the TC or LDL-C level has been acceptable although the targets were not achieved. It means that these physicians did not implant the treatment guidelines to their clinical practice and were not eager to have their patients to achieve the treatment target, or they didn’t catch up the latest guideline. About 17% physicians didn’t modify the prescription because they concerned the possible side effects induced by statin dosage adjustment might affect the drug compliance of the patients. From the patient’s aspect, 25% of patients without attaining their lipid goal preferred not increase their drug potency or dosage because of concerning the possible side effects of the more potent/higher dosage therapy. Therefore, to improve the achievement rate in Taiwan, we should educate not only the physicians, but also patients about the latest lipid guideline and the importance of guideline adherence, especially to those in the local area hospital.

In the present study, age is another significant determinant for achieving the treatment target. The result is consistent with the report of Jones, P.H., R. Nair, and K.M. Thakker [19]. In the last decade, several studies have confirmed the efficacy, safety and tolerability of HMG-CoA reductase inhibitors (statins) [20–22], and showed that elderly patients with high cardiovascular risk derive the highest benefits from statin treatment. However, statins are under-utilized in elderly patients according to the retrospective study reported by Ko, D.T., M. Mamdani, and D.A. Alter [23]. In contrast, Taiwanese physicians have done more to the lipid control in elderly patients, who have achieved the lipid target more easily than younger patients and may get higher benefit from it.

Male gender is also a determinant for target achievement. This result is in accordance with previous reports [24,25]. Singh, M., et al. conducted a retrospective study [25] to analyse the gender difference of LDL-C target achievement in secondary prevention after acute myocardial infarction over a 5-year period (2003–2007). No gender difference of lipid-lowering therapy was observed. However, females had a higher LDL than did males both in 2003 and 2007. In recent decades, more and more female patients suffer from coronary artery disease. The prognosis of female patients after acute coronary syndrome has been shown to be inferior to that of male patients [26,27]. Therefore, intensive lipid treatment should be emphasized for females to attain the lipid treatment target.

This study revealed that the target-achieved rate of lipids was significantly lower in the patients with CVD. Some previous studies have also shown similar findings [28–30]. In the World Health Organization study on Prevention of Recurrence of myocardial Infarction and StrokE (WHO-PREMISE) conducted in several developing countries, the prescription rate of statins was lower in the patients with stroke than in those with CAD [28]. In the Vascular Protection and Guideline-Oriented Approach to Lipid-Lowering registries in Canada, the LDL-C target achievement and statin use were around 10% lower in the patients with CVD than in those with CAD [29]. The CVD-CAD discrepancy in secondary prevention therapy of cardiovascular diseases can be explained in several ways. First, there are disparities of risk perception between CVD and CAD. Both patients and physicians regarded CAD as higher risk than CVD, and the risk-scoring was even lower in patients than in physicians [30]. Therefore, the risk factors management and target attainment may be dissimilar. Knowledge of risk factors for stroke and warning signs of stroke are often suboptimal [31]; only 30% of patients could recognize transient ischemic attack and minor stroke immediately after stroke in a population-based study of behaviour [32]. Second, stroke comprises heterogeneous etiologies, and the approach to management may be differential depending on the etiology. Statin for example, is recommended for use in the patients with atherosclerotic ischemic stroke or transient ischemic attack [31], but it is arguable whether statins should be routinely used in every CVD patient, including non-atherosclerotic diseases as dissection of small artery lacune [33].

Diabetic patients with existing cardiovascular diseases are considered to be in very high risk for further cardiovascular events. Previous studies have shown that patients with diabetes were more likely to have untreated or insufficiently treated dyslipidemia [34–36]. However, due to the increasing use of lipid-lowering agents, lipid goal attainment percentage in diabetic patients increased year by year in the U.S [37]. In our present study, the patient with diabetes, impaired fasting glucose, or impaired glucose tolerance is an independent determinant for target achievement, similar with patients with CAD. It means that the physicians in Taiwan have realized the importance of lipid control in high risk diabetic patients and done more effort on it.

This study has several limitations. First, this study’s patients were recruited mainly from the departments of cardiology and neurology of the teaching hospitals, unlike the case in some studies where the patient source was mainly from the general practitioners. Although the results from the present study may have the problem of generalizability of our results, in Taiwan there was little restriction of patients’ access to teaching hospitals, and most patients often continued their outpatient clinics follow-up at the same hospitals where they were hospitalized for the major diseases. Second, it is likely that patients with severe stroke with ambulation difficulty may have restricted their presentation to an outpatient clinic and hence enrolment into this study. It is possible that an overestimation of medication use in CVD patients occurred in this study. The disparity of medication between CAD and CVD patients may be even bigger than shown here. Third, we had no detailed information about patients’ compliance and duration of secondary prevention therapies, contraindication or reasons for discontinuing some medications, and lifestyle modifications. However, the clinical information about each participant was obtained from direct medical records and interviewing, and would be followed-up periodically. The validity of these data is high.

## Conclusion

Although the lipid treatment guideline adherence is improving in recent years, only 54% of the patients with cardiovascular diseases have achieved their LDL-C target in Taiwan, and the most significant determinant for this was statin therapy. However, most patients under lipid-lowering therapy were on regimens of very-low to low equipotent doses of statins. We should emphasize the importance of guideline adherence, especially the use of statin therapy, not only to physicians but also to patients.
